# Rituximab-Induced Esophageal Ulcers and Strictures Presenting as Dysphagia

**DOI:** 10.14309/crj.0000000000001939

**Published:** 2025-12-22

**Authors:** Muhammad Anas Abdulrazzak, Muhammed Yaman Swied, Obada Daaboul, Muaataz Azzawi, Abdul Monem Swied

**Affiliations:** 1 Department of Internal Medicine, Southern Illinois University School of Medicine, Springfield, IL; 2 Department of Gastroenterology, Southern Illinois University School of Medicine, Springfield, IL

**Keywords:** Rituximab, eshophageal ulcers, esophageal strictures

## Abstract

Rituximab is a monoclonal antibody that targets CD20 on B lymphocytes and is approved for the treatment of multiple hematological and autoimmune disorders. Rituximab may cause gastrointestinal side effects such as colitis, diarrhea, and bowel obstruction or perforation. Infusion-related reactions, such as throat irritation, are, however, not linked to any specific esophageal disease. In this study, we report an extremely rare case of rituximab-induced esophageal ulceration and stricture causing progressive, debilitating dysphagia, highlighting the importance of early recognition and prompt management to prevent long-term morbidity.

## INTRODUCTION

Rituximab is an anti-CD20 monoclonal antibody widely used for the treatment of autoimmune conditions, including granulomatosis with polyangiitis, rheumatoid arthritis, and the Sjögren syndrome, as well as malignancies such as B-cell lymphomas. Its suggested mechanism of action involves B-cell depletion through direct signaling, complement-mediated cytotoxicity, and antibody-dependent cellular cytotoxicity, leading to immunosuppression.^1^ While generally well tolerated, rituximab is associated with multiple side effects, most commonly being infusion reactions and neutropenia, with an incidence of at least 25% of patients.^2^ Other reported side effects include tumor lysis syndrome, hypogammaglobulinemia, infections, arrhythmias, mucocutaneous reactions, and serum sickness. Rituximab-induced colitis has been reported in the literature, but esophageal manifestations have been rarely reported.^3^

Esophagitis is a condition characterized by the inflammation of the esophageal mucosa, typically presenting with dysphagia, odynophagia, retrosternal chest pain, regurgitation, and food impaction in some patients.^4^ Common etiologies include infections (e.g., *Candida*, herpes simplex virus, cytomegalovirus), inflammatory conditions such as eosinophilic esophagitis, and direct mucosal injury from acid reflux or direct medications. Pill-induced esophagitis is well documented with agents such as tetracyclines, nonsteroidal anti-inflammatory drugs, and bisphosphonates. By contrast, rituximab-associated esophagitis is extremely rare and has been scarcely reported. We report a rare case of rituximab-induced esophageal ulcers and strictures.

## CASE REPORT

**Figure 1.**
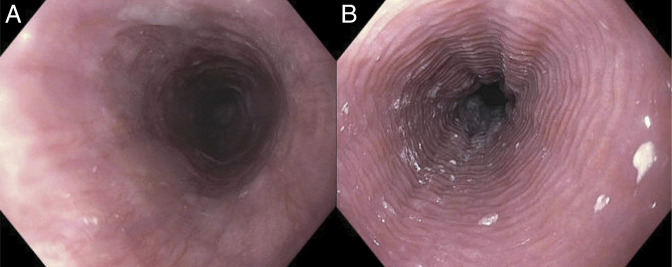
(A) EGD showing normal middle third of the esophagus. (B) EGD showing mucosal changes in the lower third of the esophagus. EGD, esophagogastroduodenoscopy.

**Figure 2.**
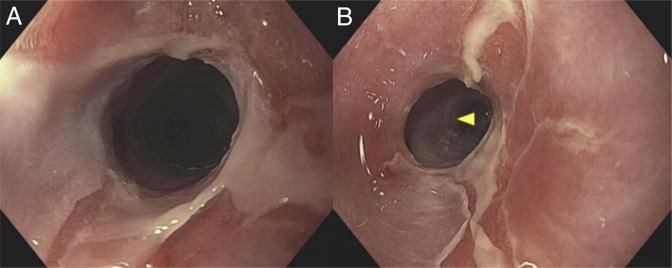
(A) EGD showing multiple esophageal ulcers in the upper third of esophagus and benign appearing esophageal stenosis at 16 cm from the incisors. (B) EGD showing multiple esophageal ulcers in the lower third of esophagus and benign appearing esophageal stenosis at 30 cm from the incisors. EGD, esophagogastroduodenoscopy.

**Figure 3.**
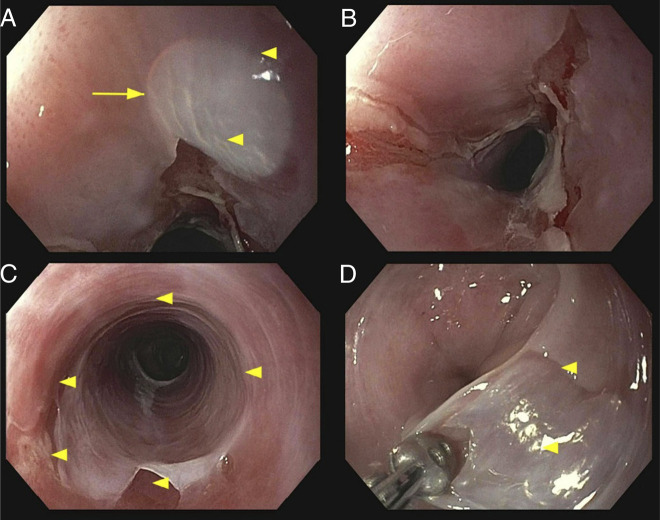
Endoscopic images from esophagogastroduodenoscopy performed before discontinuation of rituximab therapy. (A) Middle third of the esophagus – Ulcer. (B) Middle third of the esophagus – Ulcer. (C and D) Epithelium sloughing.

**Figure 4.**
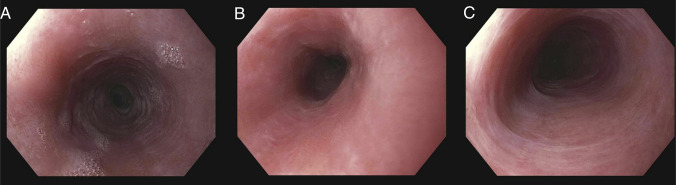
Endoscopic images of the esophagus from esophagogastroduodenoscopy performed after discontinuation of rituximab therapy, showing improvement in ulcerations and inflammation. (A) Upper third of the esophagus – Improvement in ulceration and inflammation. (B) Middle third of the esophagus – Improvement in ulceration and inflammation. (C) Lower third of the esophagus – Improvement in ulceration and inflammation.

A 53-year-old woman with stage IV follicular lymphoma presented with new-onset mild dysphagia to solids after receiving 2 cycles of induction with bendamustine and rituximab therapy. Initial esophagogastroduodenoscopy (EGD) demonstrated esophageal mucosal changes but was otherwise unremarkable (Figure 1). Biopsies were obtained and showed acute inflammation of the esophageal mucosa, but were otherwise unremarkable. The patient completed 6 cycles of induction with bendamustine and rituximab therapy, then started on maintenance rituximab every 8 weeks. The patient's dysphagia and odynophagia worsened during maintenance rituximab therapy, and a repeat EGD after cycle 2 of maintenance rituximab revealed multiple cratered and linear esophageal ulcers with active bleeding, and 2 benign-appearing esophageal stenoses at 16 and 30 cm from the incisors, which were traversed after dilation (Figure 2). The patient was started on omeprazole 40 mg twice daily. Pathology revealed active esophagitis with ulceration; however, the findings were negative for Barrett's esophagus, malignancy, and viral or fungal infection. Despite 2 months of treatment with omeprazole, symptoms persisted. Given that, omeprazole was discontinued and the patient started on vonoprazan 20 mg once daily. EGD was also performed and revealed a midesophageal stricture, as well as multiple linear, superficial esophageal ulcers with severe friability, erosions, sloughing, and ulceration (Figure 3). Pathology again showed ulceration with marked acute inflammation. Multidisciplinary discussion raised suspicion of rituximab-induced esophageal injury, prompting discontinuation of the drug after she completed 3 cycles of maintenance rituximab. Approximately 4 months after stopping rituximab, the patient experienced moderate symptomatic improvement. Follow-up EGD showed significant improvement in esophagitis and 2 benign-appearing esophageal stenoses at 20 and 30 cm from the incisors, which were successfully dilated (Figure 4). The pathology report was unremarkable. The patient was continued on vonoprazan 20 mg once daily. After 3 months, a repeat EGD showed 2 benign-appearing esophageal stenoses at 25 and 35 cm from the incisors, which were successfully dilated. Pathology was unremarkable. The patient eventually reported resolution of her dysphagia while remaining on vonoprazan 20 mg once daily. In regard to her lymphoma, after stopping maintenance rituximab for 1 year, she continued to be clinically stable, with no signs, symptoms, laboratory, or imaging findings suggestive of recurrence.

## DISCUSSION

Rituximab-induced esophageal injury is rarely reported in the literature. Harika Balagnoi et al reported a case of rituximab-induced esophagitis in a patient with stage IV B-cell lymphoma.^5^ Rituximab-induced esophagitis diagnosis was made after ruling out all potential causes, including infection and malignancy. The patient improved after rituximab was discontinued and treatment with steroids and proton pump inhibitors was initiated.

In our case, the patient developed dysphagia and multiple esophageal ulcers and strictures on EGD while on maintenance rituximab. Despite a comprehensive evaluation workup, including esophagram, multiple follow-up EGDs with biopsies, swallow studies, and testing alternative etiologies, no other cause was identified. Therefore, rituximab-induced esophagitis was suspected, prompting discontinuation of the medication to monitor the patient's response.

Several factors supported the diagnosis: the temporal association between rituximab administration and the onset of esophagitis, the absence of infectious, malignant, or autoimmune findings on biopsies, a limited response to proton pump inhibitor monotherapy, and significant improvement in symptoms and endoscopic findings upon stopping rituximab. Notably, our patient's initial EGD was negative for esophageal ulcers and strictures; however, she subsequently had worsening dysphagia and odynophagia, with new esophageal ulcers and strictures on EGD during continued rituximab therapy. This progression further supports a diagnosis of rituximab-induced esophageal injury.

Esophagitis dissecans superficialis (EDS) was also considered in our differential diagnosis. EDS is a rare, benign condition characterized by sloughing of large fragments of esophageal mucosa. It has been associated with desquamating dermatologic disorders such as pemphigus vulgaris, postendoscopic dilation, and certain medications, including NSAIDs, psychoactive agents, and bisphosphonates.^6^ However, in our patient, endoscopy revealed extensive ulceration and mucosal friability, neither of which is characteristic of EDS. Histology also did not show parakeratosis or intraepithelial splitting—features that were present in 100% and 86.1% of cases, respectively, in a large EDS case series of 41 patients.^7^ Taken together with the clear temporal association between rituximab initiation and the onset of esophagitis, these findings strongly favor rituximab-induced esophagitis over EDS as the underlying diagnosis.

While the exact mechanism and pathophysiology of this rare adverse effect remain unclear, insights can be drawn from reports of rituximab-induced colitis. It is thought to result from immune dysregulation secondary to B-cell depletion by rituximab at the level of the gastrointestinal mucosa and is the most acceptable hypothesis so far.^8^ In particular, the loss of regulatory B-cell function may halt the production of anti-inflammatory cytokines, such as IL-10, and thereby promote unchecked T-cell activation, infiltration, and mucosal inflammation; however, this theory has not yet been tested or confirmed.

Although esophageal injury caused by rituximab is rarely documented in the literature, clinicians should be aware of this possible side effect and promptly conduct a gastrointestinal evaluation in patients who develop dysphagia and odynophagia while receiving rituximab therapy. Routine screening of all individuals receiving rituximab may not be necessary; however, increased awareness can help ensure timely referrals and interventions if symptoms like dysphagia or odynophagia occur.

In conclusion, rituximab was likely the cause of the esophageal ulcers and strictures in our patient, which improved after rituximab was discontinued. Our case highlights the importance of considering the possibility of rituximab-induced esophageal injury in patients presenting with dysphagia while taking rituximab therapy.

## DISCLOSURES

Author contributions: MA Abdulrazzak wrote the first draft of the manuscript and edited the final version after receiving input from the other authors. MY Swied reviewed and edited the first and final draft of the manuscript and is the article guarantor. O. Daaboul reviewed and edited the final draft of the manuscript. M. Azzawi reviewed the final draft of the manuscript. AM Swied reviewed and approved the final draft of the manuscript.

Financial disclosure: None to report.

Previous presentation: This case has been presented at the ACG Annual Meeting October 25–29, 2025; Phoenix, AZ.

Informed consent was obtained for this case report.

## ABBREVIATIONS:

EDS: Esophagitis dissecans superficialis
EGD: Esophagogastroduodenoscopy
